# Weak coordination among petiole, leaf, vein, and gas‐exchange traits across Australian angiosperm species and its possible implications

**DOI:** 10.1002/ece3.1860

**Published:** 2015-12-29

**Authors:** Sean M. Gleason, Chris J. Blackman, Yvonne Chang, Alicia M. Cook, Claire A. Laws, Mark Westoby

**Affiliations:** ^1^ Department of Biological SciencesMacquarie UniversityNorth RydeNSW2109Australia; ^2^ Water Management Research UnitUSDA–ARSFort CollinsColorado80526; ^3^ Hawkesbury Institute for the EnvironmentUniversity of Western SydneyRichmondNSW2753Australia; ^4^ CSIRO AgricultureLB 59NarrabriNSW2390Australia

**Keywords:** Angiosperms, CO_2_ assimilation, gas exchange, plant hydraulics, stomatal conductance, trait coordination

## Abstract

Close coordination between leaf gas exchange and maximal hydraulic supply has been reported across diverse plant life forms. However, it has also been suggested that this relationship may become weak or break down completely within the angiosperms. We examined coordination between hydraulic, leaf vein, and gas‐exchange traits across a diverse group of 35 evergreen Australian angiosperms, spanning a large range in leaf structure and habitat. Leaf‐specific conductance was calculated from petiole vessel anatomy and was also measured directly using the rehydration technique. Leaf vein density (thought to be a determinant of gas exchange rate), maximal stomatal conductance, and net CO_2_ assimilation rate were also measured for most species (*n* = 19–35). Vein density was not correlated with leaf‐specific conductance (either calculated or measured), stomatal conductance, nor maximal net CO_2_ assimilation, with *r* ^2^ values ranging from 0.00 to 0.11, *P* values from 0.909 to 0.102, and *n* values from 19 to 35 in all cases. Leaf‐specific conductance calculated from petiole anatomy was weakly correlated with maximal stomatal conductance (*r* ^2^ = 0.16; *P* = 0.022; *n* = 32), whereas the direct measurement of leaf‐specific conductance was weakly correlated with net maximal CO_2_ assimilation (*r* ^2^ = 0.21; *P* = 0.005; *n* = 35). Calculated leaf‐specific conductance, xylem ultrastructure, and leaf vein density do not appear to be reliable proxy traits for assessing differences in rates of gas exchange or growth across diverse sets of evergreen angiosperms.

## Introduction

Water conductance through plant xylem and vapor phase conductance from leaves must be coupled to comply with the principles of mass balance (Lomonosov 1760). Therefore, the capacity of xylem to transport water should be aligned with maximal rates of transpiration (Brodribb and Feild 2000; Sperry 2000; Sterck and Schieving 2011). At the leaf level, hydraulic capacity of the petiole (petiole conductance) should be coordinated with the total size of the leaf lamina (Sack et al. 2003). In addition to leaf size, we might expect that species with high petiole conductance relative to leaf size to exhibit faster water vapor loss per unit leaf area than species with lower petiole conductance. For example, if we were to compare leaves of similar size, we might expect higher rates of transpiration from leaves with higher petiole conductance. This expectation has been supported by strong correlations between gas exchange and leaf‐specific hydraulic conductivity measured in stems (Brodribb and Feild 2000; Santiago et al. 2004). It is also supported by strong correlations between hydraulic conductance measured within the leaf and rates of gas exchange across diverse taxa (Brodribb et al. 2007; Brodribb and Jordan 2008), but also within a single species (Brodribb and Jordan 2011). In contrast, other studies have found poor coordination between these traits (angiosperms in Brodribb and Holbrook 2006; Maherali et al. 2006; angiosperms in Brodribb et al. 2007; Fichot et al. 2011). Nearly all these studies have measured the maximal capacity for photosynthesis or transpiration, using gas‐exchange apparatus on wild‐grown plants under favorable conditions (well illuminated and well hydrated). It is this maximal capacity for gas exchange that is expected to be coordinated with the capacity of the hydraulic system to deliver water.

If the maximal supply of water through a petiole is coordinated with the size of leaves and the rate of vapor loss from leaves, we might also expect coordination between the capacity of vein networks to supply water to the leaf surface and the rate of water vapor loss from the leaf surface (Brodribb et al. 2007; Boyce et al. 2009; Brodribb and Jordan 2011). This is because more numerous and efficient leaf veins reduce the distance liquid water must travel from vein endings to the points of evaporation within the leaf lamina, wherever that is presumed to be (Brodribb et al. 2007; Noblin et al. 2008; Zwieniecki and Boyce 2014), although this relationship is likely shifted by leaf thickness (Noblin et al. 2008; Sack et al. 2013). Taken together, we might expect coordination among the maximal capacity of petioles to transport water, the density of vein systems to distribute that water within the leaf, and the measured rates of gas exchange from the leaf surface.

Clearly, there are good a priori reasons to expect strong hydraulic coordination, and it has been reported when comparing across ferns, gymnosperms, and angiosperms. The question we ask here is whether this pattern of coordination is maintained across morphologically and ecologically diverse angiosperm species? We first tested this idea by looking solely at the anatomical and morphological coordination between petioles, leaf size, and leaf venation. To do this, we asked whether the maximal capacity of petioles to transport water (i.e., petiole hydraulic capacity) is well correlated with the area of the leaf supported by petioles? Next, we asked, do species with small leaves and high petiole conductance (i.e., potentially more water delivered per unit leaf area) possess higher vein densities to distribute this water within the leaf (i.e., is *calculated* leaf‐specific conductance correlated with vein density?). We expand this question further to examine possible coordination between vein density and *measured* leaf‐specific conductance. We then asked whether leaf‐specific conductance (calculated or measured) or vein density is correlated with maximal stomatal conductance and CO_2_ assimilation. Lastly, we asked whether climate differences among sites (precipitation or aridity) modify the relationship between stomatal conductance and calculated leaf‐specific conductance?

## Materials and Methods

### Sites and species

Species were studied at five sites in eastern Australia, spanning a wide range of mean annual temperature (MAT; 10.0–21.3°C), mean annual potential evapotranspiration (PET; 847–1839 mm), aridity (MAP/PET; 0.99–0.31), and latitude (42.4° to 18.3°S). We named these sites by their relative latitude (temperate, subtropical, tropical) and relative aridity (wet, dry, monsoonal), for example, “Temperate‐Dry”. Vegetation at all sites were late successional forest communities with well‐developed understories. At each site between five and nine of the most common evergreen angiosperms were sampled, representing 13 different plant families (Supporting information, Trait Data).

Our aim in taking hydraulic, anatomical, and gas exchange measurements was to obtain quantities that characterized the species and the sites, with a view to consider the coordination of traits *across species*. If hydraulic coordination is an important outcome of natural selection, we should expect covariation among hydraulic traits across species' mean values (our focus here), whereas correlated variation within species would reflect an ability of species to make plastic or physiological adjustment in a coordinated way.

### Sampling for anatomical measurements

For each species, three to six large shoots (ca 1–2 m in length) were cut from the sunny portion of the canopy of five individual plants. Five to ten sun leaves (petioles intact) were cut from these shoots and preserved in a formalin‐acetic‐alcohol (FAA) solution for later anatomical work, described below. Collections were made using an extendable pruning pole, or in some cases, a hydraulic elevated lift platform. Months of peak temperature and aridity were avoided to ensure nonstressed leaf conditions. Leaf collection dates were as follows: Temperate‐Dry (October 2012), Subtropical‐Wet (March 2012), Subtropical‐Dry (May 2012), Subtropical‐Arid (August 2012), and Tropical‐Monsoonal (June 2012).

### Gas‐exchange measurements

Gas‐exchange measurements were undertaken in March 2010 at the Temperate‐Dry site, October 2010 at the Subtropical‐Wet site, June 2009 at the Subtropical‐Dry site, April 2009 at the Subtropical‐Arid site, and November 2009 at the Tropical‐Monsoonal site. Canopies were accessed from the ground or using ladders. When individual plants exceeded the reach of ladders (ca 5 m), smaller individuals were chosen. One or two fully expanded sun leaves from five individual plants were chosen for measurement during morning hours (0900–1100). Excessively dry and excessively wet periods were avoided during measurements. Maximal stomatal conductance (*g*_s_) and net CO_2_ assimilation (*A*_max_) were measured using a portable photosynthesis system (Model 6400xt; LI‐COR Biosciences, Lincoln, NE). Temperature, reference CO_2_, and VPD were kept within narrow ranges for all measurements (23–27°C, 388–402 ppm, 1.9–2.1 kPa). Photosynthetically active radiation (PAR) was increased in a step‐wise fashion for each species until maximal rates of gas exchange were achieved (900–1800 *μ*mol·quanta·m^−2^·s^−1^), but also to ensure that sun‐sensitive species were not over saturated.

### Climate Data

Climate data for the collection sites were taken from the Australian Bureau of Meteorology and summarized to reflect climate during the peak growth seasons at each site (Specht and Brouwer 1975; Prior et al. 2004). Precipitation (PPT), temperature, vapor pressure deficit of the atmosphere (VPD), and aridity (VPD/PPT) were calculated as mean annual values, as well as during the months of peak growth. December and January were considered the months of peak growth in the temperate and subtropical sites (Specht and Brouwer 1975), whereas January and February were considered peak growth months at the tropical monsoonal site (“Topical‐Monsoonal”) (Prior et al. 2004). Data were downloaded from individual weather stations maintained by the Australian Bureau of Meteorology and represent in most cases at least 50 years of observations at (or near) each site.

### Vessel anatomy and calculation of xylem‐ and leaf‐specific conductance

Three representative leaves for each species were removed from the FAA preservative solution (sampling described above). Petioles, midribs, 2nd‐order, and minor veins were first hand sectioned near the midpoints of petioles or midribs and set in 2% agarose. Samples ~20 microns thick were transverse‐sectioned using a vibrating microtome (Leica VT1000S; Heidelberger, Germany), stained with 5% toluidine blue solution, and photographed (Nikon DXM1200F; Tokyo, Japan) under proper magnification for the respective section (Olympus BX50; Tokyo, Japan). Three images each of petiole, midrib, 2nd‐order, and minor vein cross‐section anatomy were obtained for each species. Using IMAGEJ open‐source software (Schneider et al. 2012), the total number of vessels as well as all vessel diameters were measured (along the long and short axes) within these delineated areas from properly scaled images. For petioles and midribs, areas of xylem, which contained 30–90 vessels, were used as subsamples to estimate the total number and average diameter of all vessels within these major vein structures.

Conductance through each vessel was calculated using the formula given in Lewis and Boose (1995) for elliptical transverse sections. The conductance through the sampled areas of xylem was calculated (disregarding pit membrane and end‐wall resistances) by summing hydraulic conductance for all conduits within each sample area. Calculated xylem‐specific conductance (*K*_X_) was determined by dividing the calculated conductance for each cross‐section by the area of xylem present in each cross‐section (A). Calculated hydraulic conductance of petioles and xylem‐specific conductance were calculated for each petiole (3 replicates·species^−1^), and each vein order (3 vein orders × 3 replicates·species^−1^). Calculated leaf‐specific hydraulic conductance (*K*_L_) was calculated by dividing petiole conductance by the leaf area supported by the petiole, that is, the leaf size was scanned prior to sectioning (Epson Perfection V30 scanner, Seiko Epson, Nagano, Japan). These estimates of xylem‐specific conductance correlate with *measured* branch xylem‐specific conductivity (*r* ^2^ = 0.48; *P* < 0.001) (Gleason et al. 2012) (Fig. S1, Supporting information). Correlation between these two independent methods suggest that acceptable estimates of hydraulic functioning can be obtained by scaling vessel traits up to cross‐sections and petioles, even when measurements are taken at different locations in the plant, on different individuals, and across different years.

### Direct measurement of leaf‐specific conductance

In addition to calculating leaf‐specific conductance (*K*_L_‐calc) from vessel anatomy and leaf size, leaf‐specific conductance was also measured directly using the rehydration method (*K*_L_–meas) (Brodribb and Cochard 2009). One large branch (1–2 m length) was cut from the sun‐lit portion of the each canopy (five individual plants per species) during the early morning when plants were relatively hydrated. Branches were then allowed to dry down slightly (Ψ between −0.4 and −1.0 MPa) in a sheltered and shaded location. We thus assume that leaf xylem experiences a negligible loss of conductance between 0 and −1.0 MPa, which is strongly supported by percent loss of conductance (PLC) curves for these (Blackman et al. 2014). Branches were then placed in a plastic bag for ca 30 min to allow for equilibration of xylem water potential. Branches were then removed from the plastic bag, and two directly adjacent leaves or small shoots (ca. 10 cm length) were marked for measurement. One leaf/shoot was then cut free from the branch using a razor blade, wrapped in a moist paper towel to arrest evaporation, and immediately placed in a pressure chamber for measurement of leaf/shoot water potential. The other leaf/shoot was cut free from the branch under water and attached to a water‐filled 1‐mm‐diameter silicon tube while remaining submerged. The other end of the silicon tube was placed in a water‐filled 50‐ml beaker resting on a laboratory balance (Sartorius CP225D; Göttingen, Germany). The rate of rehydration into the leaf was then measured as the mass loss from the balance (logged every 2 s), that is, the water transferred into the leaf through the petiole. Leaf‐specific conductance was calculated as the water loss rate from the balance normalized by the pressure gradient (assumed equal to leaf water potential), leaf area, and corrected for water viscosity. The initial rehydration rate was used for all measurements (ca the first 5–10 sec of rehydration).

### Vein density measurements

Three representative leaves for each species were sampled and preserved in FAA solution. We used the leaf clearing and vein quantification protocol described in Scoffoni et al. (2011). Prior to leaf clearing, 3rd‐order and minor veins located centrally in the leaf lamina were exposed by removing the epidermis and top layers of mesophyll with a razor blade. Leaves were “cleared” in 1 mol·L^−1^ KOH, which removed nonlignified tissues, leaving the venation network bare, but intact. Cleared leaves were stained with Safranine‐O and counter stained with fast‐green. Leaves were mounted onto transparencies and scanned at 1000 dpi using a flatbed scanner. Leaf area and the length of 1st‐ and 2nd‐order veins were measured using IMAGEJ open‐source software (Schneider et al. 2012). The length of the 3rd‐order and minor veins were measured from the exposed sections from images taken at 4× and 10× magnification, respectively, using a camera mounted to the light microscope. The 3rd‐order and minor vein densities were calculated as the total length of each vein order (mm) divided by the area of each image (mm^2^). Major vein density was determined as the sum of the 1st‐, 2nd‐, and 3rd‐order vein densities and the total vein density as the sum of all vein order densities. Thus, vein densities were assessed on one leaf for three individual plants (*n* = 3) per species.

### Analyses and transparency

Plant traits were plotted against one another, and correlation coefficients were calculated from linear models to assess the covariation between pairs of plant traits. Power analyses were run on each correlation to evaluate the statistical capacity of each test to detect linear trends, assuming they exist (i.e., possibility for type II error). The “pwr” package for R was used for these analyses (Cohen 1988). Site‐specific differences were compared by fitting each individual site with a separate standard major axis (SMA) trendline and comparing slope and intercept coefficients (intercepts were tested only when sites shared a common slope) using the “smatr” package for R (Warton et al. 2006). The “shift” test in this R package was also used to test for significant dispersion of species from different sites along a common axis (rather than having different regression coefficients) (Warton et al. 2006). In these cases, mean site axis scores were calculated for each site and regressed against the climate data to determine whether climate differences were aligned with the observed across‐site species' differences.

To assess the relative influence of xylem area and xylem‐specific conductance (calculated from petiole anatomy) on whole‐petiole conductance, the relative variance was partitioned as in Onoda et al. (2011). Briefly, calculated petiole conductance can be expressed as: *K*_P_ = *K*_X_ *·A*, where *K*_X_ = the xylem‐specific conductivity and A = the cross‐sectional area of xylem. After log transformation, the equation can be written as: log(*K*_*P*_) = log(*K*_X_) + log(*A*). In this form, the total variance in log(*K*_P_) is equal to cov(log*K*_P_, log*K*_X_) + cov(log*K*_P_, log*A*). In other words, these two covariance terms measure the fractional contributions from *K*_X_ and *A* to the total variance in *K*_P_. The “var” and “cov” functions in R were used for this analysis (R Core Team 2014).

We provide the entire dataset used in these analyses as Microsoft Excel and CSV text files, for easy exporting to data analysis programs (Tables S2 & S3, Supporting information). To add transparency to our analyses, we also provide the R scripts used to generate all figures included in this report (R Scripts 1–3, Supporting information). These scripts can be used seamlessly with the CSV dataset in the R environment (R Core Team 2014).

## Results

### Question 1: is the calculated conductance of petioles closely aligned with leaf size?

**Figure 1:**
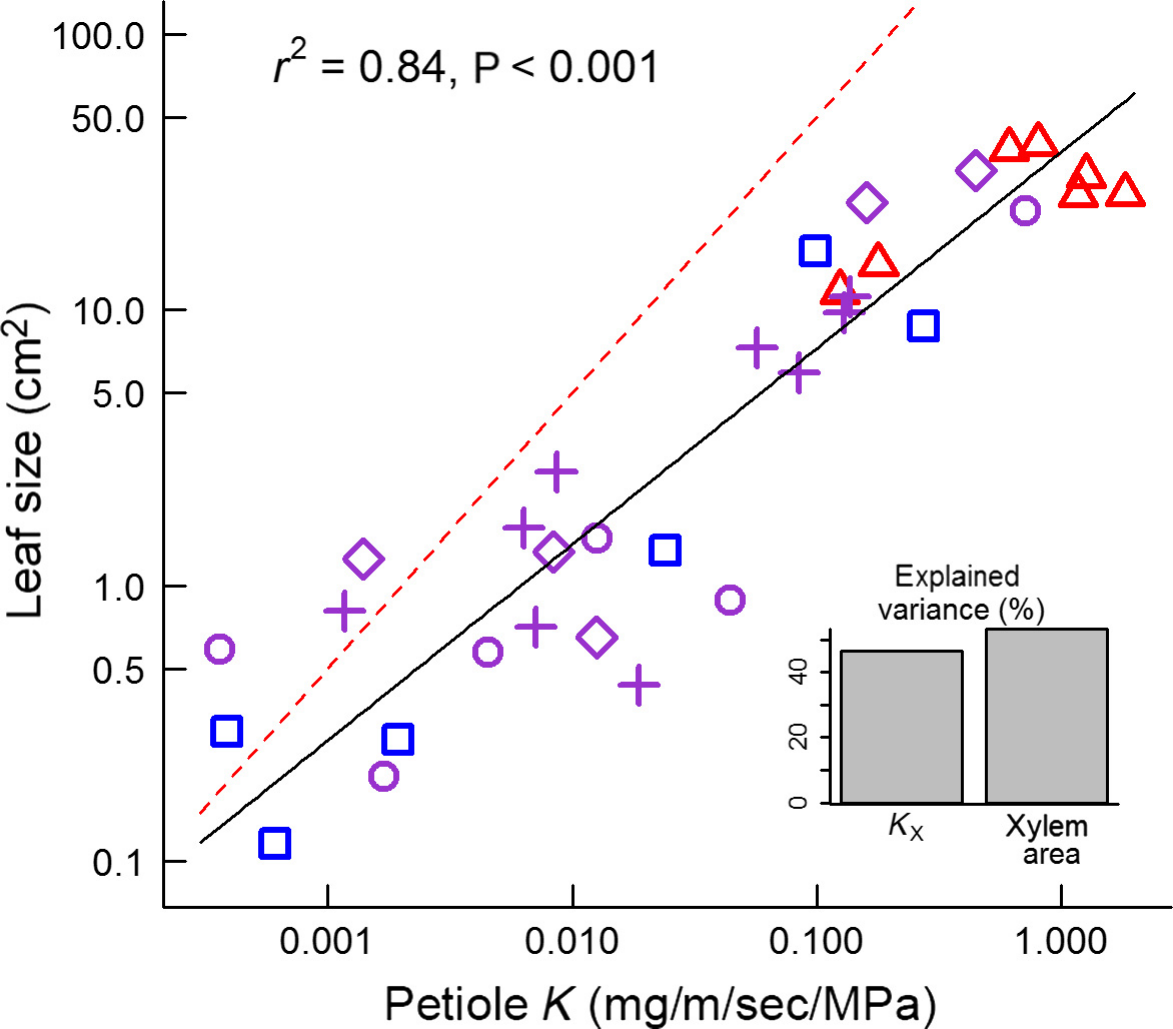
Relationship between leaf size and calculated petiole conductance (petiole *K*) across all sites and species. Sites are denoted by the following symbols: Tropical‐Monsoonal (▵), Subtropical‐Wet (○), Subtropical‐Dry (♢), Subtropical‐Arid (+), and Temperate‐Dry (□). Symbol color denotes temperature: temperate = blue, subtropical = magenta, tropical = red. Standard major axis trendline (solid black) and broken red line show departure in fitted exponent (0.71) from proportional changes in leaf size. Individual symbols represent species' means.

Calculated petiole conductance was a strong predictor of leaf size across the species in this study (*r* ^2^ = 0.84; *P* < 0.001), but leaf size increased less than proportionately with calculated petiole conductance (leaf size ~ petiole conductance scaling exponent = 0.71) (Fig. 1). Calculated petiole conductance has two components: calculated xylem‐specific conductance (*K*_X_) and the total cross‐sectional area of xylem present in the petiole (xylem area A). These components both contributed strongly to variation in calculated petiole conductance (*K*_X_ 46%, A 53%, bottom‐right insert in Fig. 1). Calculated xylem‐specific conductance in petioles varied over two orders of magnitude across the species in this study (from 0.053 to 6.11 Kg·m^−1^·s^−1^·MPa^−1^) and greatly facilitated increases in leaf area (Fig. 1; *r* ^2^ = 0.64; *P* < 0.001). The less‐than‐proportional increase in leaf size with calculated petiole conductance (Fig. 1) indicates that species with larger leaves may have access to more water (through the petiole) per unit leaf area than smaller leaves, across a given pressure gradient. Although larger leaves appeared to have a better supply of water (higher leaf‐specific conductance), should we also expect leaves with higher calculated leaf‐specific conductance to have a denser vein network to distribute that water and higher rates of stomatal conductance and CO_2_ assimilation?

### Question 2: does leaf‐specific conductance of petioles correlate with vein density measurements?

**Figure 2:**
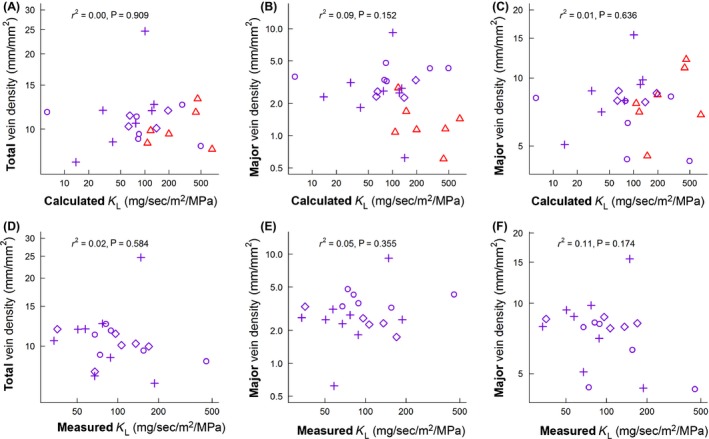
Relationship between leaf‐specific conductance and vein density. Leaf‐specific conductance calculated from petiole vessel anatomy (calculated *K*_L_) versus total vein density (A), major vein density (B), and minor vein density (C). Panels D–F show the same comparison as above, but using leaf‐specific conductance values measured using the rehydration technique (measured *K*_L_). Sites are denoted by the following symbols: Tropical‐Monsoonal (▵), Subtropical‐Wet (○), Subtropical‐Dry (♢), and Subtropical‐Arid (+). Symbol color denotes temperature: subtropical = magenta, tropical = red. Individual symbols represent species' means. The number and identity of species on each plot may differ depending on the availability of measurements (Supporting information, Trait Data).

Leaf‐specific conductance calculated from petiole anatomy was not correlated with total vein density (*r* ^2^ = 0.00; *P* = 0.909), major vein density (*r* ^2^ = 0.09; *P* = 0.152), nor minor vein density (*r* ^2^ = 0.01; *P* = 0.636) (Fig. 2A–C). This suggests that the density of leaf veins to distribute water was not coordinated with the anatomical capacity of petioles to deliver water, at least for the species and habitats examined here. Directly measured leaf‐specific conductance was also not correlated with total vein density (*r* ^2^ = 0.02; *P* = 0.584), major vein density (*r* ^2^ = 0.05; *P* = 0.355), nor minor vein density (*r* ^2^ = 0.11; *P* = 0.174) (Fig. 2D–F). Statistical power of all vein density correlations was sufficient to detect a trend (*α *= 0.05) with *r* ^2^ values as low as 0.16 (calculated *K*_L_) or 0.20 (measured *K*_L_). This further suggests that neither the measured nor calculated rates of maximal leaf conductance (as estimated from anatomy and the rehydration technique) are well coordinated with the density of the vein network, and certainly supports the conclusion that vein density does not have sufficient predictive power to be used as a proxy trait for leaf‐specific conductance across evergreen angiosperms. Furthermore, these two estimates of leaf‐specific conductance (calculated vs. measured) were also not correlated with one another (*r* ^2^ = 0.06; *P* = 0.306) (Fig. S2, Supporting information), suggesting that the size and number of vessels in petioles are poor predictors of the rehydration rate (at least for the species reported here) and that other sources of resistance in leaves (e.g., mesophyll resistance) may be better predictors of leaf rehydration rate than petiole hydraulic capacity and lamina area (Scoffoni et al. 2014).

### Question 3: does leaf‐specific conductance or vein density correlate with measured rates of stomatal conductance and co_2_ assimilation?

**Figure 3:**
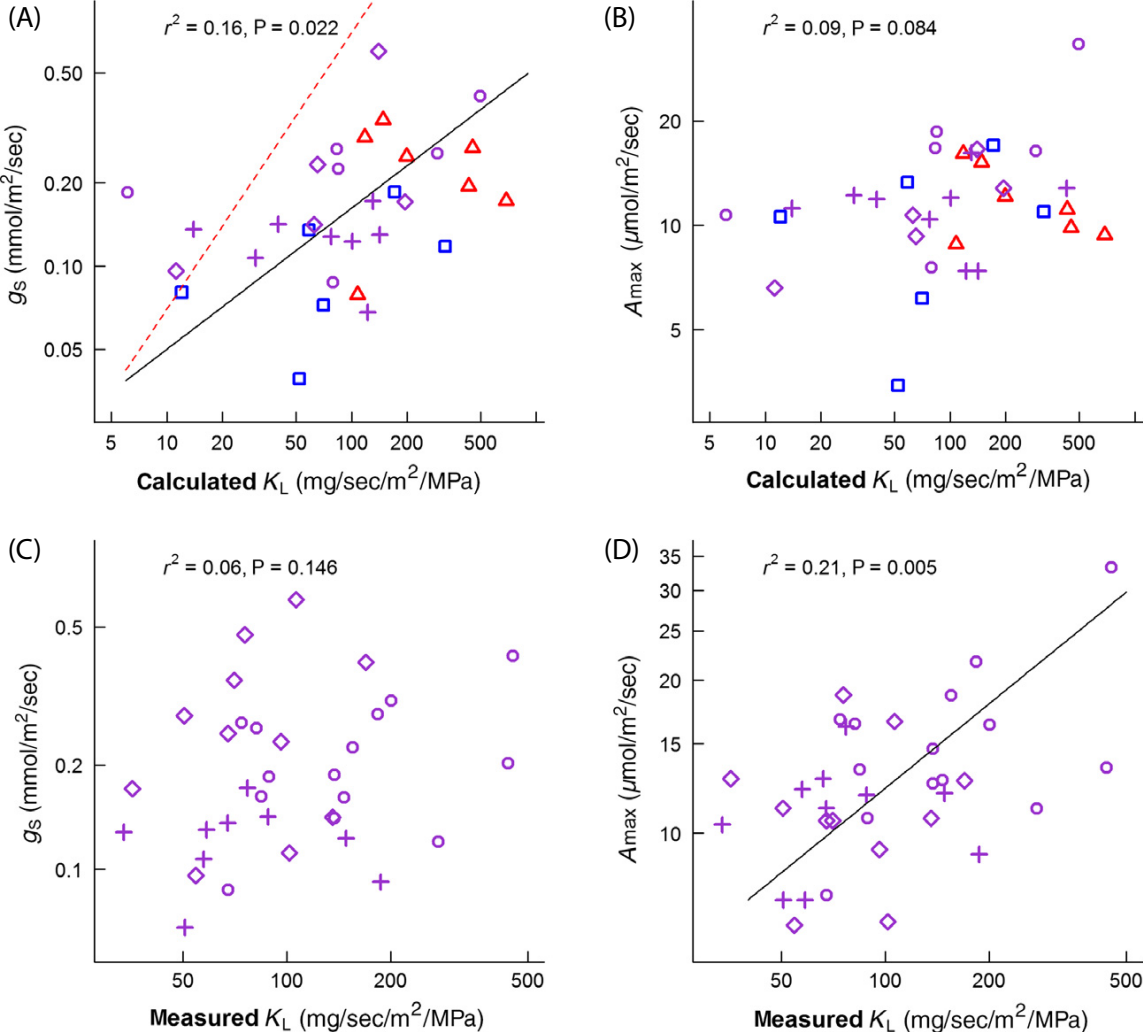
Relationships between leaf‐specific conductance measurements and gas exchange. Relationship between leaf‐specific conductance calculated from petiole vessel anatomy (calculated *K*_L_) and stomatal conductance (*g*_s_) (A), and maximal CO_2_ assimilation rate (*A*_max_) (B). Panels C and D show the same comparison as above, but using leaf‐specific conductance values measured using the rehydration technique (measured *K*_L_). Sites are denoted by the following symbols: Tropical‐Monsoonal (▵), Subtropical‐Wet (○), Subtropical‐Dry (♢), Subtropical‐Arid (+), and Temperate‐Dry (□). Symbol color denotes temperature: temperate = blue, subtropical = magenta, tropical = red. Standard major axis trendline (solid black) and broken red line departure in fitted exponent from proportional changes in *g*_s_ (A). Individual symbols represent species' means. The number and identity of species on each plot may differ depending on the availability of measurements (Supporting information, Trait Data).

**Figure 4:**
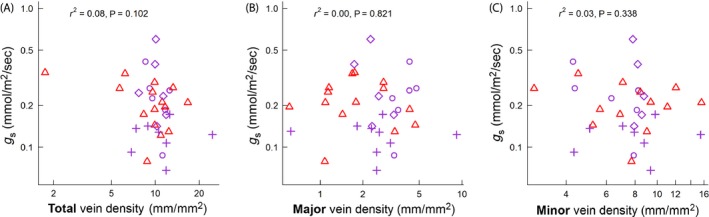
Relationship between stomatal conductance (*g*_s_) and total vein density (A), major vein density (B), and minor vein density (C). Sites are denoted by the following symbols: Tropical‐Monsoonal (▵), Subtropical‐Wet (○), Subtropical‐Dry (♢), and Subtropical‐Arid (+). Symbol color denotes temperature: subtropical = magenta, tropical = red. Individual symbols represent species' means. The number and identity of species on each plot may differ depending on the availability of measurements (Supporting information, Trait Data).

Leaf‐specific conductance calculated at the petiole was significantly, but weakly, correlated with stomatal conductance (*r* ^2^ = 0.16; *P* = 0.022) (Fig 3A). The *g*_s_ ~ *K*_L_–calc scaling exponent in this case (0.511) was significantly <1 (*P* < 0.001), indicating that maximal stomatal conductance did not scale proportionately with the capacity of petioles to deliver water, that is, the red line in Fig 3A. The relationship between calculated leaf‐specific conductance and maximal CO_2_ assimilation rate was also poor (*r* ^2^ = 0.09; *P* = 0.084) (Fig. 3B). Directly measured leaf‐specific conductance (rehydration technique) was not correlated with stomatal conductance (*r* ^2^ = 0.06; *P* = 0.146), but was correlated with maximal rates of CO_2_ assimilation (*r* ^2^ = 0.21; *P* = 0.005) (Fig 3C,D). No measure of vein density was significantly correlated with maximal stomatal conductance (Fig 4) or CO_2_ assimilation across the range of vein densities spanned by the angiosperms in this study. Values of *r* ^2^ and *P* for all six correlations ranged from 0.00 to 0.11 and 0.821 to 0.062, respectively. Statistical power of all gas exchange ~ vein density correlations was sufficient to detect a trend (*α *= 0.05) with *r* ^2^ values as low (or lower) as 0.12 in all cases.

### Question 4: do differences in precipitation or climate aridity modify the relationship between stomatal conductance and leaf‐specific conductance?

The poor relationships between leaf‐specific hydraulic capacity and gas exchange across the species in this study (Fig. 3) might suggest sources of resistance beyond the xylem (e.g., in soil) are more important than resistance in the petiole. For example, it has been suggested that differences in soil water potential, operating leaf potential, and evaporative demand may shift the relationship between hydraulic capacity and leaf gas exchange (Sack et al. 2013). To assess this idea, we compared the site‐specific slopes and intercepts taken from the *g*_s_ ~ *K*_L_–calc relationship. We used *K*_L_–calc in these analyses, rather than *K*_L_–meas (rehydration) because the *g*_s_ ~ *K*_L_–meas correlation was non‐significant and *K*_L_–calc was quantified for more species and sites. Considering that sites differ markedly in temperature, precipitation, and aridity, we hypothesized that different climates might engender different slope or intercept coefficients. However, the coefficients relating *g*_s_ to *K*_L_–calc did not differ significantly across sites. Rather, sites were significantly (*P* = 0.006) separated along a common *g*_s_ ~ *K*_L_–calc trendline. Species from higher precipitation and lower aridity tended to operate at higher stomatal conductance and *K*_L_–calc compared to species from sites with lower precipitation and higher aridity. Specifically, mean site scores along a common slope (i.e., the distance between sites along the fitted trendline in Fig. 3A) were positively correlated with growth season precipitation (*r* ^2^ = 0.89; *P* = 0.016) and marginally negatively correlated with growth season aridity (PPT/VPD) (*r* ^2^ = 0.69; *P* = 0.082). These relationships with climate were the main driver of the relationship between *K*_L_–calc and stomatal conductance. Relationships within sites were weak or absent (Fig. 3A). Additionally, mean site values of *K*_L_–calc were positively correlated with precipitation (*r* ^2^ = 0.80; *P* = 0.040) and negatively correlated with aridity (*r* ^2^ = 0.84; *P* = 0.028) during the growth season, meaning that species with high leaf‐specific conductance tend to favor sites with high water availability and low aridity.

## Discussion

### Why might leaf‐specific conductance be poorly correlated with gas exchange?

It is likely that coordination exists among xylem, vein, and gas exchange across taxa from bryophytes through ferns to gymnosperms and angiosperms (Brodribb and Feild 2000; Brodribb et al. 2007; Brodribb and Jordan 2008). So why should these relationships break down within the subset of evergreen angiosperms examined here? We propose three main reasons why these traits might appear poorly coordinated when compared across species and habitats: (1) methodological difficulties with gas exchange and estimates of conductance result in much measurement error, (2) differences in habitat and plant structure that alter the forces driving water though the xylem, and (3) biological reasons for the lack of coordination, other than habitat and plant structure.

### Methods and climate

Maximal rates of gas exchange are difficult to measure, and especially so under field conditions. In addition to the relatively large standard deviations among measurements, stomatal conductance and CO_2_ assimilation also vary markedly throughout the day and season (Long et al. 1996; Long and Bernacchi 2003; Prior et al. 2004), which is why maximal gas exchange is measured during near‐optimal conditions, as we have done here. Similar to the measurements reported here, previous studies supporting coordination performed one‐time measurements of gas exchange on wild plants (Santiago et al. 2004; Brodribb et al. 2007; Brodribb and Jordan 2008, 2011). It is also possible that gas exchange measurements made in the field may not align with maximal hydraulic capacity if the water potential of the xylem at the time of measurement was sufficiently low to engender significant loss of conductance. However, we, as well as the studies mentioned above, have minimized this possibility by taking measurements on well‐hydrated plants during morning hours and under conditions conducive to maximal rates of gas exchange (temperature, VPD, radiation, ambient CO_2_). Therefore, there appears no reason to expect methodological errors to differ across studies, although it is possible that these methods have resulted in important errors in all studies which have used them. Importantly, we examined hydraulic coordination across species, that is, using species' mean values, because we are interested in coordination arising from natural selection, not correlations associated with subtle differences in light or water availability among individual leaves. Reporting correlations between paired hydraulic traits across individuals, rather than species, would not have addressed the evolutionary importance of hydraulic coordination.

Another potential source of error is how we *calculated* petiole and leaf‐specific conductance from vessel diameter measurements. As such, we assume other components of xylem resistance (e.g., intervessel pit resistance) vary little across species. This is a particularly important shortcoming considering that pit and pit‐membrane ultrastructure varies widely across species, even within a single genus (Lens et al. 2011, 2013). However, our estimates of xylem‐specific conductance in petioles are correlated with *measured* xylem‐specific conductance in branches (*r* ^2^ = 0.48; *P* < 0.001) (Gleason et al. 2012) (Fig. S1, Supporting information). Thus, two independent estimates (one measured, one calculated) of xylem‐specific conductivity are in good agreement, even though they were taken from different structures (branches vs. petioles), measured in different years, and on different individuals. In other words, there is good evidence for the evolutionary coordination between branch and petiole xylem‐specific conductance, but little evidence for the coordination among petiole conductance, vein density, and gas exchange. Furthermore, our direct measurements of leaf‐specific conductance using the rehydration technique also failed to support the idea of close coordination between leaf conductance, vein density, and gas exchange across evergreen angiosperms.

The supply of water per unit leaf area depends not only on the resistance through the petiole, but on driving force for water transport (water potential difference between the leaf and soil) as well as resistances throughout the leaf, through the entire plant, and in the soil. If unmeasured sources of resistance were different between species (e.g., differences in path‐length from root to vein or from vein ending to the points of evaporation), or if the driving force for water transport differed substantially from species to species, we should not expect close across‐species coordination between gas‐ and liquid‐phase conductance measured only in leaves. Nevertheless, the studies that have reported tight relationships between hydraulic supply and gas exchange, similarly, have not measured whole‐path resistance or scaled leaf conductance to account for differences in driving force across species (Ψ_leaf_, Ψ_soil_). Although this suggests that results reported here and elsewhere are not likely to differ in this respect, it does not rule out the possibility that sources of resistance beyond the petiole and leaf, as well as differences in driving force, have introduced meaningful errors in all cases.

The pressure gradient driving the movement of water through the plants (water availability and evaporative demand) also varied widely in this study. Both climate and rates of gas exchange covaried weakly with calculated leaf‐specific conductance, suggesting that sites with high water availability tended to favor species with high leaf‐specific conductance and somewhat greater rates of gas exchange. However, these differences in climate actually contributed to the correlation between calculated leaf‐specific conductance and leaf gas exchange (Fig. 3A), rather than weakening it.

### Possible biological reasons for poor coordination

Photosynthesis might saturate at low vein densities (ca <2 mm·mm^−2^) if CO_2_ assimilation becomes either biochemically limited (e.g., Rubisco‐mediated carboxylation capacity; Vcmax) or becomes limited by the maximal rates of CO_2_ conductance through stomata and mesophyll tissue, rather than by the supply of water delivered via the vein network (Boyce and Zwieniecki 2012). Our data are in rough agreement with this idea, considering that all but two of our species exhibited vein densities >8 mm·mm^−2^. However, this does not explain why carboxylation capacity would not have evolved to meet both the supply of CO_2_ and water. Similarly, it does not explain why evolution would not “wind back” stomatal conductance and hydraulic supply if there were not sufficient carboxylation capacity, that is, it does not explain the poor coordination between maximal hydraulic and gas‐exchange capacities.

Photosynthesis is a temperature‐sensitive process, often with little variation in operating temperature across species that vary enormously in their distribution (Cunningham and Read 2002, 2006; Helliker and Richter 2008) and canopy position. Transpirational cooling can be a large factor in controlling leaf temperature, along with differences in leaf angle, reflectance, and exposure to direct radiation (Medina et al. 1978; Leuzinger and Körner 2007; Helliker and Richter 2008). If transpiration provided greater benefits for cooling, rather than carbon assimilation, we might expect a disconnect between hydraulic capacity and photosynthetic rates. However, hydraulic capacity would still be expected to be coordinated with stomatal conductance, yet this coordination was largely absent in this study (Fig. 3A).

Xylem‐specific conductance often decreases daily (Meinzer et al. 2008), seasonally (Prior and Eamus 2000; Brodribb et al. 2002), and during drought (Zhou et al. 2013), although this idea has recently been challenged for tree species (Cochard and Delzon 2013). Thus, we might expect natural selection to favor built‐in redundancy in hydraulic systems so that loss of xylem conductance does not unnecessarily limit gas exchange, similar to redundancy systems that have been suggested for angiosperm leaves (Wagner 1979; Bohn et al. 2002; Sack et al. 2008) and other biological networks (Tononi et al. 1999). Any variation in xylem redundancy across species would result in poor alignment between hydraulic capacity and maximum stomatal conductance. Furthermore, we might also expect species exhibiting marked resistance to hydraulic dysfunction to exhibit greater redundancy (Scoffoni et al. 2011), and therefore, poor coordination between liquid‐ and gas‐phase conductive capacities.

Lastly, lowering resistance in the xylem may have a benefit in its own right, independent of its link to gas exchange. Increasing xylem conductance will result in proportional decreasing tension in branch and leaf xylem. Providing that soil water potential is high (wet soil), increasing xylem‐specific conductance should not necessarily subject the hydraulic network to embolism risk. In fact, if resistance in the xylem tissue could be completely eliminated (i.e., *K*_L_ = ∞), leaf water potential would equilibrate to soil water potential. Such a strategy in wet habitats might allow plants to avoid carbon costs associated with building dense embolism‐resistant xylem, refilling embolized vessels (Brodersen et al. 2010), or osmotic adjustment in leaves (Hummel et al. 2010). Although this idea has been discussed previously (Whitehead 1998; Zach et al. 2010; Gleason et al. 2013), we feel it remains an interesting possible explanation for the marked variation in xylem‐specific conductivity among angiosperm species.

## Conclusion

Strong coordination has been previously reported among petiole, veins, and stomata when comparing across species from bryophytes to angiosperms. This coordination is clearest when species with vein densities below 5–10 mm·mm^−2^ are considered; many of these low densities coming from ferns and gymnosperms. Understanding this coordination has undoubtedly improved our understanding of water use and photosynthesis, and has provided an impetus for new theoretical work (Noblin et al. 2008; Boyce et al. 2009; Boyce and Zwieniecki 2012; Brodribb et al. 2013; Feild and Brodribb 2013; Sack et al. 2013). Yet, it is equally important to understand why coordination among these traits is often weak or absent across species, as reported here and elsewhere (angiosperms in Brodribb and Holbrook 2006; Maherali et al. 2006; angiosperms in Brodribb et al. 2007; Fichot et al. 2011; Walls 2011). This indicates the evolutionary drivers linking hydraulics and gas exchange are not completely understood.

We note many instances when we might not expect close coordination between hydraulic capacity and gas exchange capacity. However, the counter‐argument to most of these scenarios is that although other factors come into play (e.g., resistance elsewhere in the plant and soil), from a least‐cost perspective, evolution would not be expected to result in over‐designed hydraulics, nor in under‐designed gas exchange.

Coordination between maximum hydraulic supply and gas exchange is often assumed in process‐based growth models. Furthermore, anatomical data are now being used as proxy measures for rates of gas exchange and plant growth. Our data from evergreen angiosperms, as well as published and unpublished data from others, do not support these assumptions. For example, the largest hydraulic dataset to date fails to support the idea of coordination between leaf‐specific conductance measured in branches and leaf gas exchange across evergreen and deciduous angiosperm species (Xylem Functional Traits Database; https://www.try-db.org/TryWeb/Home.php) (Fig. S3, Supporting information). Thus, our results here are not the only instance of poor support for this idea. As such, we suggest that careful consideration of these results should be given before using anatomic measurements such as vessel dimensions, calculated rates of conductance, and vein density as proxy measurements for rates of stomatal conductance, CO_2_ assimilation, or growth.

## Conflict of Interest

None declared.

## Data Accessibility

The complete dataset used in this report, as well as the R scripts used to generate the figures, are available as Supporting Information.

## Supporting information


**Figure S1.** Relationship between xylem‐specific conductance calculated from petiole vessel anatomy (petiole *K*_S_) and xylem‐specific conductivity measured in branch sapwood, as reported in Gleason et al. (2012).

**Figure S2.** Relationship between measured and calculated leaf‐specific conductance for some of the species reported in this study (species for which both measurements were taken).

**Figure S3.** Relationship between leaf‐specific conductance (branches) and stomatal conductance across 217 individual observations, representing 67 families and 181 species.

**Table S1.** Species' means for all traits.

**Table S2.** CSV text file of all data used in these analyses.
**Table S3.** MS Excel file of all data used in these analyses.

**R Script S1.** R script for re‐drawing Fig. 1 in the R environment.

**R Script S2.** R script for re‐drawing Fig. 2 in the R environment.

**R Script S3.** R script for re‐drawing Fig. 3 in the R environment.

**R Script S4.** R script for re‐drawing Fig. 4 in the R environment.
