# Supplementary figures and images for: Weak coordination among petiole, leaf, vein, and gas‐exchange traits across Australian angiosperm species and its possible implications
Source: Ecol Evol. 2015 Dec 29;6(1):267–78. doi: 10.1002/ece3.1860 (PMC4716519; doi:10.1002/ece3.1860)

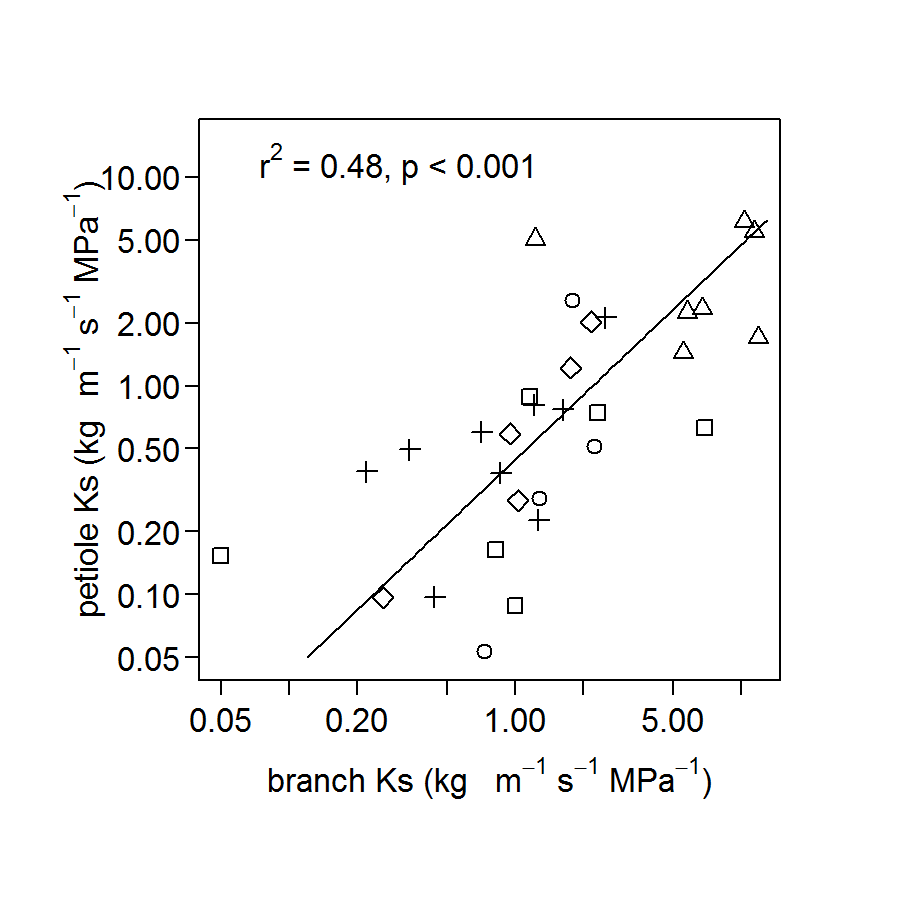

Supplement: Supplementary file 1 — Figure S1. Relationship between xylem‐specific conductance calculated from petiole vessel anatomy (petiole K S) and xylem‐specific conductivity measured in branch sapwood, as reported in Gleason et al. (2012). [file ECE3-6-267-s001.tiff]

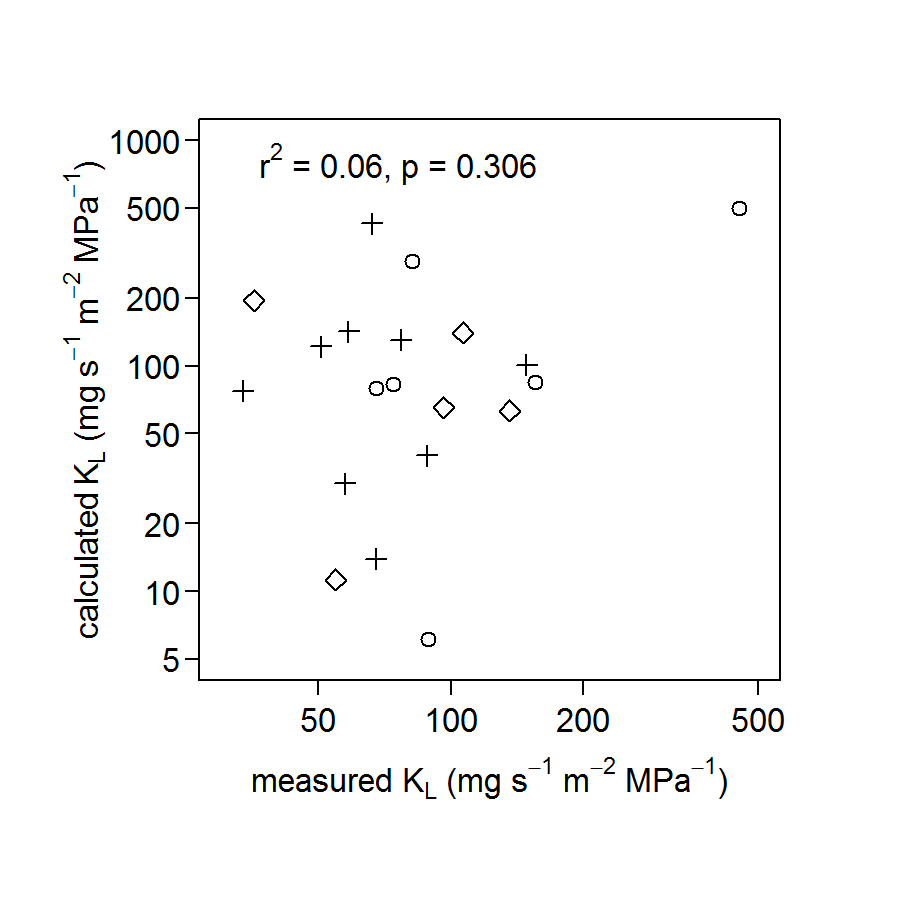

Supplement: Supplementary file 2 — Figure S2. Relationship between measured and calculated leaf‐specific conductance for some of the species reported in this study (species for which both measurements were taken). [file ECE3-6-267-s002.tiff]

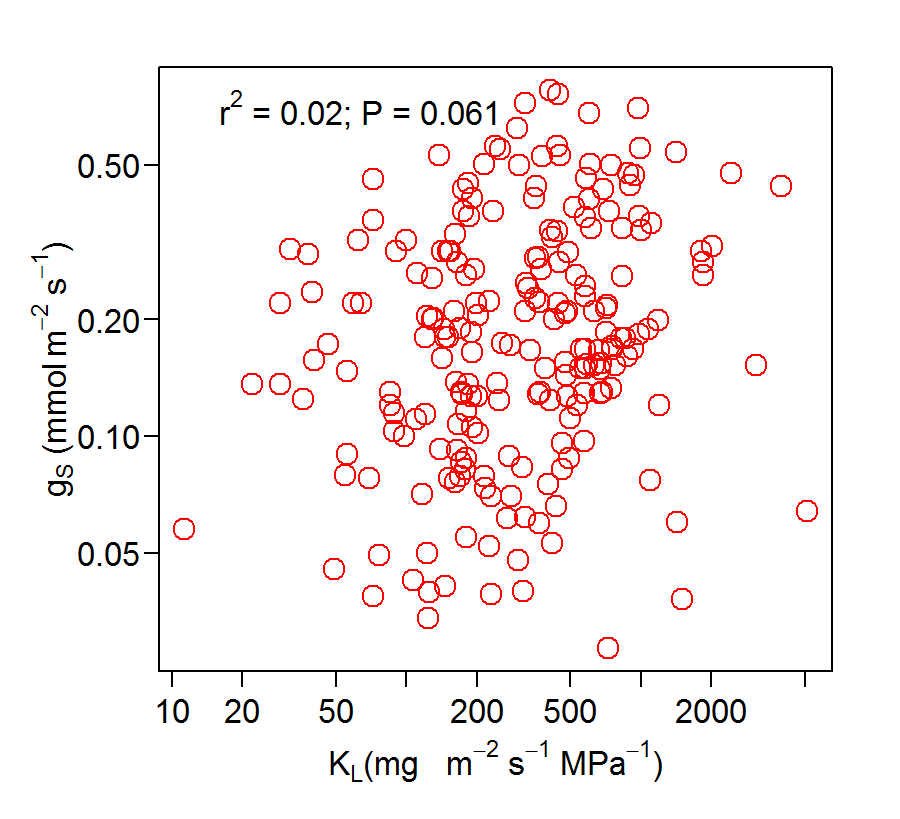

Supplement: Supplementary file 3 — Figure S3. Relationship between leaf‐specific conductance (branches) and stomatal conductance across 217 individual observations, representing 67 families and 181 species. [file ECE3-6-267-s003.tiff]
