# Supplementary material for: Weak coordination among petiole, leaf, vein, and gas‐exchange traits across Australian angiosperm species and its possible implications
Source: Ecol Evol. 2015 Dec 29;6(1):267–78. doi: 10.1002/ece3.1860 (PMC4716519; doi:10.1002/ece3.1860)

**Supplemental Information 1.**

**Fig. S1.** Relationship between xylem-specific conductance calculated from petiole vessel anatomy (petiole K_S_) and xylem-specific conductivity measured in branch sapwood, as reported in Gleason et al. (2012). This relationship suggests that reliable estimates of xylem-specific conductivity can be estimated from vessel anatomy, even when measurements are taken at different locations in the plant (branches vs petioles), on different plants, and in different years. Sites are denoted by the following symbols: Tropical-Monsoonal (△), Subtropical-Wet (○), Subtropical-Dry (◇), Subtropical-Arid (🞢), and Temperate-Dry (□).


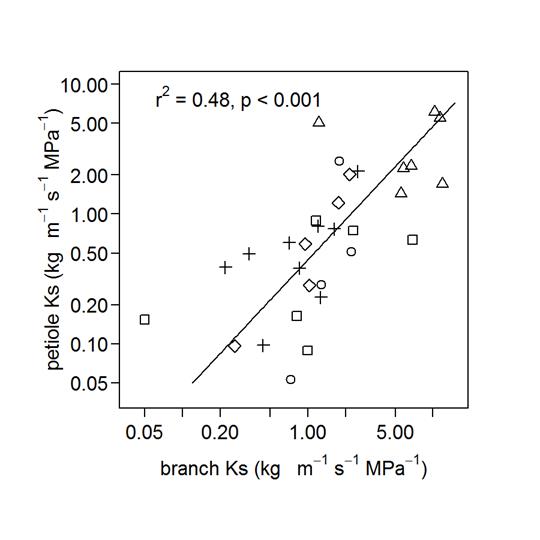


**Fig. S2.** Relationship between measured and calculated leaf-specific conductance for some of the species reported in this study (species for which both measurements were taken). Sites are denoted by the following symbols: Subtropical-Wet (○), Subtropical-Dry (◇), Subtropical-Arid (🞢).


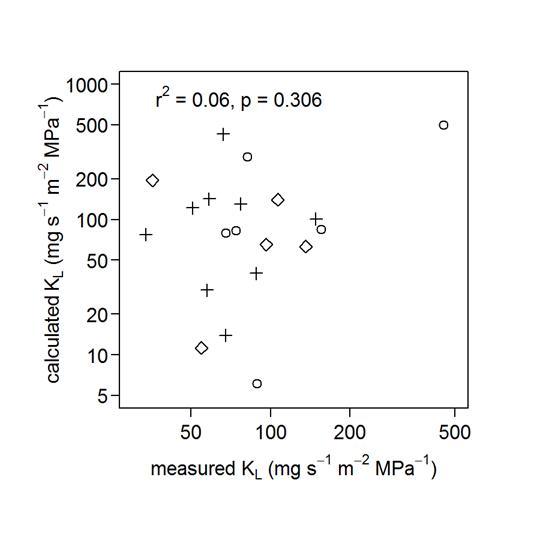


**Fig. S3** Relationship between leaf-specific conductance (branches) and stomatal conductance across 217 individual observations, representing 67 families and 181 species. Data obtained from the Xylem Functional Traits Database; XFT (<https://www.try-db.org/TryWeb/Home.php>).


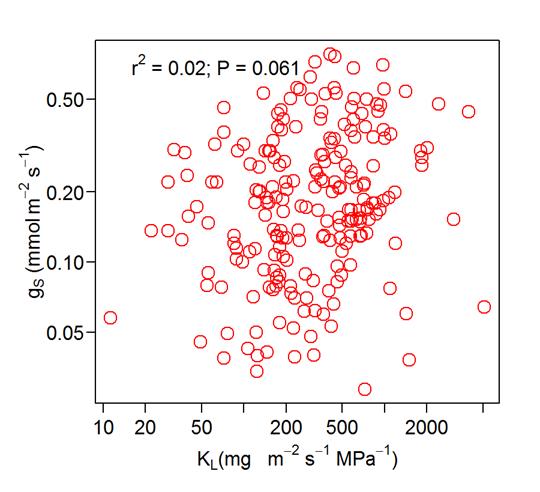

Supplement: Supplementary file 4 [file ECE3-6-267-s004.docx]
